# Antifungal persistence: Clinical relevance and mechanisms

**DOI:** 10.1371/journal.ppat.1013456

**Published:** 2025-09-11

**Authors:** Rui Wang, Jingnan Lv, Liang Chen, Yanan Zhao, Hong Du

**Affiliations:** 1 Department of Clinical Laboratory, The Second Affiliated Hospital of Soochow University, Suzhou, China; 2 MOE Key Laboratory of Geriatric Diseases and Immunology, The Second Affiliated Hospital of Soochow University, Suzhou, Jiangsu China; 3 Department of Pharmacy Practice, School of Pharmacy and Pharmaceutical Sciences, University at Buffalo, Buffalo, New York, United States of America; 4 Key Laboratory of Alkene-Carbon Fibres-Based Technology and Application for Detection of Major Infectious Diseases, Suzhou, China; University of Georgia, UNITED STATES OF AMERICA

## Abstract

Antifungal persistence is the phenomenon that occurs when a subpopulation of fungal cells can survive in the presence of high concentrations of antifungal drugs, which is different from the concepts of antifungal resistance and tolerance. Fungal persisters are not mutants but phenotypic variants of normal cells, entering a dormant state with low metabolism and proliferation. Previous studies have shown that antifungal persistence may lead to therapeutic failure, as well as chronic or recurrent fungal infections in clinical settings. This review provides a comprehensive overview of antifungal persistence covering its definition, distinctions from other related concepts, detection methods, and molecular mechanisms of formation. Importantly, we discuss relevant *in vivo* experiments and clinical observations to assess clinical relevance of antifungal persistence.

## Introduction

In 1944, Joseph Bigger observed a small subpopulation of *Staphylococcus* spp. survived after penicillin treatment, and first proposed the concept of “persisters” [1]. Antifungal persistence had not been well studied for many years, until LaFleur et al. found Amphotericin B (AmB)-tolerant persisters in *Candida albicans* biofilms [2]. Today, it has been identified in various *Candida spp.* and even in filamentous fungi, both in vitro and in vivo [3,4]. However, antifungal persistence is often interchangeable with tolerance and is easily mistaken for resistance or heteroresistance, leading to confusion and reduced comparability between studies.

Invasive fungal diseases (IFD) are associated with high mortality and are a huge public health menace, caused by pathogenic yeast such *C. albicans* or filamentous fungi such as *Aspergillus fumigatus* [5,6]. Currently, only three primary classes of agents are used to treat IFD: polyenes (such as AmB, which induces ergosterol sequestration); azoles (such as fluconazole, which induces the inhibition of ergosterol biosynthesis; and echinocandins (such as caspofungin, which induces the inhibition of β-1,3-glucan synthase). When antifungal therapy fails, the development of drug resistance is a common explanation and clinicians may change the regimen to another drug or use combination therapy [7,8]. However, in some cases, even though devoid of resistance, the clinical treatment still failed, leading to breakthrough or recurrent infections [9–11]. One possible explanation is antifungal persistence, given its ability to survive lethal drug exposure without an increase of resistance level [12]. Previous studies have supported this point of view, and some drugs have been developed to eradicate persisters [13,14].

The definition, detection, and mechanisms of Antifungal persistence and its clinical relevance will be discussed in depth in this review.

## Overview of antifungal persistence

### 1. Definition

According to a recent consensus statement of antibiotic persistence [15], antifungal persistence can be defined as a population-level phenomenon in which only a subpopulation survives high-dose treatment with antifungal drugs. Fungal persisters are dormant variants of wild-type cells that are tolerant to antifungal drugs, surviving high dosage of antifungals that exceed the minimal inhibitory concentration (MIC) [12,16].

### 2. Persistence versus resistance, tolerance, and heteroresistance

**Fig 1:**
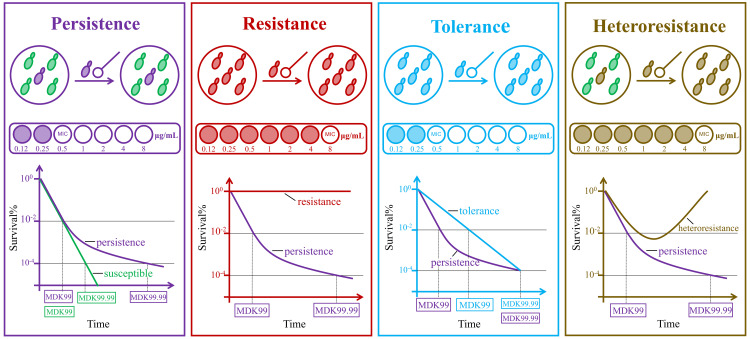
Persistence versus resistance, tolerance, and heteroresistance. First of all, the progeny of persisters will still divide into persistence subpopulations and non-persistence subpopulations, while resistance and tolerance can be stably inherited to their progeny. In antifungal susceptibility testing, the MIC of persistence and tolerance do not increase, while resistance and the resistant population of heteroresistance show a high level of MIC. In time-kill assay, antifungal persistence is characterized by biphasic-killing curve, which represents the similar MDK_99_ as a susceptible strain. However, MDK_99.99_ is substantially higher for a persistent strain than the MDK_99.99_ for a susceptible strain. Concentrations and timescales are chosen for illustration purposes only. Abbreviations: MIC, minimal inhibitory concentration; MDK_99_, minimum duration of treatment that kills 99% of the population; MDK_99.99_, minimum duration of treatment that kills 99.99% of the population.

There are fundamental differences between antifungal persistence and resistance (Fig 1). Firstly, antifungal persistence represents the ability of a small subpopulation to survive under fungicidal drugs, while resistance represents the ability of the entire population to not only survive but also replicate during drug exposure. Secondly, antifungal persistence is determined by the characteristic biphasic-killing curve in the time-kill assay, without an increase in MIC. However, resistance is characterized by increased MIC in the antifungal susceptibility testing. Thirdly, antifungal persistence is non-heritable, although the progeny of persisters may still divide into persistent subpopulations and non-persistent subpopulations. In contrast, resistance is linked to specific molecular mechanisms and can be stably inherited by progeny [15,17–19].

In research on molecular mechanisms, “antifungal persistence” and “tolerance” are often used interchangeably, as both describe the survival of fungal cells under high doses of antifungal drugs without an increase in MIC. However, these terms reflect distinct phenomena. Antifungal persistence is a heterogeneous trait, where only a small fraction of the population (generally <<1%) is able to survive drug exposure, while the majority of non-persistent cells are rapidly killed by the treatment. This duality reveals that persistence represents a heterogeneous rather than uniform response, for which “hetero-tolerance” may be a more accurate designation. In time-kill assay, the minimum duration for killing (MDK) can be used to detect persistence. Persistent strain and susceptible strain take similar time to reach the minimum duration of treatment that kills 99% of the population (MDK_99_), because initially most of the persistent strain population is susceptible to antifungal treatment and is eliminated in a manner similar to a susceptible strain. Over time, the persistent subpopulation emerges and maintains viability, leading to longer minimum treatment durations required for 99.99% population eradication (MDK_99.99_) [15,17,20,21] (Fig 1). For fungicidal agents, MDK_99_ and MDK_99.99_ can also be used to detect fungicidal tolerance, such as detecting the tolerance of *Cryptococcus neoformans* to AmB [22]. However, because antifungal tolerance is most evident with fungistatic drugs (fungistatic tolerance), measuring cell death is less relevant. *In vitro* assay that can quantify both susceptibility (measured as MIC at 24 h) and growth properties at 48 h (the fraction of growth or supra-MIC growth) was recommended to use [23].

Antifungal heteroresistance refers to the coexistence of a small population of resistant cells (usually <<1%) and a large population of susceptible fungal cells (Fig 1). Antifungal heteroresistance was first been found in *C. neoformans* and *Cryptococcus gattii*, and was considered as an intrinsic property of these species, associating with disomic chromosomes [24–27]. In multiple murine infection models, Heteroresistant strains can lead to increased fungal burden and reduced treatment efficacy [28,29]. Moreover, a recent study found that antifungal heteroresistance causes prophylaxis failure and facilitates breakthrough *Candida parapsilosis* infections in patients during allogeneic hematopoietic cell transplantation [30]. Antifungal heteroresistance differs fundamentally from antifungal persistence, as it involves replicative capacity under drug pressure accompanied by an elevated MIC. The population analysis profile assay is used as gold standard for detecting heteroresistance [31].

### 3. Detection

Currently, antifungal persistence is mainly identified in *Candida*-associated biofilms [2,32,33]. It is difficult to detect persisters in planktonic cells, which is different from bacteria [12,16]. One possible explanation is that the formation of antifungal persistence depends on adhesion (see Section 3 below: Adhesion plays a key role in antifungal persistence formation) [34]. Amphotericin B which belongs to polyenes class, due to its potent fungicidal activity and the rarity of resistance [35–37], has been widely studied and shown to induce antifungal persistence in *Candida* spp. biofilms and Trichosporon spp. Reports on echinocandin-associated persistence are primarily focused on intracellular *Nakaseomyces glabratus* (also called *Candida glabrata*), which internalized by macrophage [38,39]. Notably, the paradoxical effect, also known as the “Eagle effect”, often observed in echinocandins may confused with antifungal persistence, as the drugs show reduced activity *in vitro* at higher concentrations [40,41]. Given that azoles are fungistatic to most yeast pathogens, it can be challenging to determine whether azole tolerance is associated with persistence or the inherent properties of the drug itself [17]. When it comes to filamentous fungi, before detecting azole persistence, it is recommended to determine killing effects first. A paper proposes that a drug that can kill ≥90% of spores in 24 h (MDK90 ≤ 24 h) of all isolates of a given species should be considered fungicidal [42].

For a long time, characteristic biphasic-killing curve observed in the time-kill assay and survival rate evaluated by colony-forming unit (CFU) counting were the only ways to identify and evaluate antifungal persistence *in vitro* [15,20]. These methods, derived from the definition, are the basis for research of antifungal persistence, but they lack the sensitivity and resolution required for precise identification. To solve these problems, live/dead staining method can be applied because of its ability to distinguish live/dead cells, which reflects the essence of persisters that survive exposure to fungicides. For example, using a scanning confocal microscope, fluorescein diacetate can stain live cells green, whereas propidium iodide (PI) stains dead cells red, enabling the visual identification of persisters [2,12,43]. Further, combining live/dead staining method with flow cytometry, antifungal persistence can be detected more accurately in real-time and in a high-throughput manner. *TDH3*, a gene encoding glyceraldehyde-3-phosphate dehydrogenase, has higher expression in stationary phase cells than in log phase. By PI staining and GFP labeling TDH3, persisters characterized as PI(−) and GFP(+) can be detected and sorted by flow cytometry [44]. In another research, a SYTOX-based flow cytometry assay was developed to evaluate the persistence level, demonstrating high predictive accuracy compared to the traditional CFU counting method [39].

However, the aforementioned detection methods can only detect persistence after its formation. To enable earlier and real-time detection of persister cells, several biomarkers have been identified. Under stress conditions, cells with high expression of heat-shock protein 12 (Hsp12) survived better than those with normal expression, suggesting that Hsp12 is a possible molecular marker of persistence [45]. In a recent research, Sps1(encoded by gene CNAG_00848, named stationary-phase specific indicator 1) detected by fluorescence-activated cell sorting and ergothioneine (provide protection in reactive oxygen species) evaluated by Raman spectroscopy can both reflect the level of *Cryptococcus* persistence, both *in vitro* and *in vivo* [3].

## Clinical relevance of antifungal persistence

### 1. Potential outcome of antifungal persistence

In clinical practice, antifungal susceptibility testing is commonly used to distinguish drug-resistant and sensitive isolates, so as to predict the outcome of antifungal treatment and guide clinical medication [46]. However, antifungal tolerance and persistence are often overlooked [17,47]. Recent research has linked antifungal persistence to recurrent infections, offering insights into why drug-sensitive strains may display only limited or even no response to antifungal therapy.

Antifungal persistence has been observed *in vivo* in a few studies using animal models [3,39,48]. For example, echinocandin-tolerant persisters were found in the mouse model of *N. glabratus* systematic infection [39]; and AmB-tolerant persisters were found in *Cryptococcus* pulmonary infection mouse model [3]. Another investigation utilizing a murine model of systemic *C. albicans* infection demonstrated that although antifungal treatment achieved substantial eradication of fungal cells, persistent pathogen reservoirs were consistently detected in gallbladder tissue during long-term monitoring [48].

A study in the *Galleria mellonella* mini-host model of *A. fumigatus* infection found that fungal burden of the larvae infected with persistent isolates was significantly higher than non-persistent isolates after voriconazole treatment [4]. Similarly, in *N. glabratus* systemic infection mouse model, mice infected with high persistent strains had significantly higher fungal burden and echinocandin-resistance mutation rate than those low persistent strains [39]. LaFleur and colleagues reported that isolates from cancer patients with long-term *C. albicans* oral colonization displayed significantly higher levels of persistence compared to those from short-term carriers [13]. Moreover, in a clinical study on the efficacy of fluconazole, it was found that the clinical isolates from patients with persistent candidemia showed high tolerance to azole drugs compared to those who could be effectively treated, which may be related to antifungal persistence [47]. In general, it can be hypothesized that a reduction in therapeutic efficacy or clinical failure can be linked to individuals being infected with high-persistent strains, though further research is needed to confirm this relationship.

### 2. Treatment targeting antifungal persistence

In antibiotic persistence, persisters are often recalcitrant to multiple antibiotics [49,50], which has been observed with fungal persisters as well. For example, *C. albicans* persister biofilms are recalcitrant to AmB and broad-spectrum antimicrobial agent chlorhexidine [2]; *N. glabratus* persisters are recalcitrant to echinocandins and AmB [38]. Therefore, clinical management of antifungal persistence presents substantial treatment challenges.

In a study of *Cryptococcus* persisters, an antidepressant drug sertraline demonstrated satisfactory anti-persistence effect both *in vitro* and *in vivo* [3]. In addition, some new drugs specifically targeting persisters have been developed. *Lavandula angustifolia* essential oil, which can provoke intracellular reactive oxygen species (ROS) accumulation, has been found to successfully eradicate caspofungin-triggered *Candida auris* biofilm persisters [14]. In a mouse model of vulvovaginal candidiasis, polymethacrylates was more effective at eradicating persisters and induced lower levels of persistence on their own, compared to conventional antifungals such as clotrimazole and nystatin [51]. Similarly, a new azole, VT-1161, not only eradicated AmB-tolerant persisters in biofilms, but also prevented the adhesion of *C. albicans* to human cells *in vitro* [52]. gH625-M, a peptide analogue of gH625 monomer, has been found to successfully eradicate persister-derived biofilms of **C. albicans* in vitro*, both alone and in combination with fluconazole and 5-fucytosine [53]. An overview of treatment targeting antifungal persistence is presented in Table 1.

**Table 1:** Overview of treatment targeting antifungal persistence.

Compound	Target species	Infection type	Persistence phenotype	Reference
Sertraline	*Cryptococcus neoformans*	Pulmonary cryptococcosis mouse model	AmB-tolerant	[3]
*Lavandula angustifolia* essential oil	*CRYPT auris*	Biofilm in vitro	Caspofungin-tolerant	[14]
Polymethacrylates	*Candida albicans*	Vulvovaginal candidiasis mouse model	\	[51]
VT-1161	*Candida albicans*	Biofilm in vitro	AmB-tolerant	[52]
gH625-M	*Candida albicans*	Biofilm in vitro	\	[53]

## Mechanisms of antifungal persistence formation

### 1. Dormant state promotes antifungal persistence

Dormant state exhibits low metabolic activities, including decreased adenosine triphosphate (ATP), transcription and translation levels, in which the growth rate is zero [46]. It has been well studied that dormant state reduces the exposure of antibiotic targets and helps bacterial cells tolerate a variety of antibiotics, a process linked to the toxin-antitoxin system [18,54]. Dormant cells have been observed in various cases of antifungal persistence, such as in AmB-tolerant *C. albicans* biofilms and in a mouse model of pulmonary cryptococcosis [3,33]. In this chapter, we will focus on the mechanisms by which the dormant state promotes antifungal persistence.

**Fig 2:**
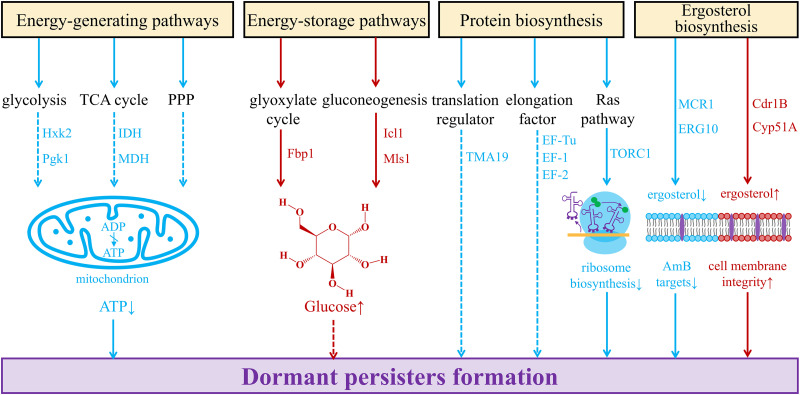
Dormant state promotes antifungal persistence. Energy-generating pathways are down-regulated in persisters, including glycolysis, TCA cycle and PPP. It has been proven that reduced ATP level is directly related to the formation of persistence. Meanwhile, energy-storage pathways are up-regulated in persisters, including glyoxylate cycle and gluconeogenesis. In general, protein biosynthesis is down-regulated in persisters, including translation regulator TMA19, elongation factor EF-Tu, and TORC1 involved in Ras pathway. Ergosterol biosynthesis is down-regulated in AmB-tolerant perisisters with decreased AmB targets, and it is up-regulated in voriconazole-tolerant Aspergillus fumigatus persisters with improved cell membrane integrity. Solid lines: proven in persisters. Dashed lines: likely to occur in persisters. Blue color: down-regulated. Red color: up-regulated. Abbreviations: TCA cycle, tricarboxylic acid cycle. PPP, pentose phosphate pathway. ATP, adenosine triphosphate. TORC1, target of rapamycin complex 1.

Through proteomics, Li and colleagues found that the major energy-generating pathways were down-regulated in AmB-tolerant *C. albicans* biofilm persisters, including glycolysis, tricarboxylic acid (TCA) cycle and pentose phosphate pathway (PPP). Specifically, phosphoglycerate kinase (Pgk1) and hexokinase-2 (Hxk2) involved in glycolysis, together with isocitrate dehydrogenase (Idh2) and malate dehydrogenase (Mdh1) involved in TCA cycle are down-regulated [33]. Glycolysis is an important and highly conserved energy-generating pathway, independent of oxygen [55]. In bacteria, the addition of glycolysis intermediates can kill aminoglycoside-induced persisters [56], but such phenomenon has not been observed in fungi. TCA cycle provides nicotinamide adenine dinucleotide as a substrate for the oxidative respiratory chain, generating a large amount of ATP [57]. A recent study has found that reduced ATP synthesis due to limited respiration may be a key mechanism of AmB-tolerant *Cryptococcus neoformans* persisters formation [3]. Conversely, key enzymes in glyoxylate cycle, named isocitrate lyase (Icl1) and malate synthase (Mls1), together with rate-limiting enzyme in gluconeogenesis named fructose-1,6-bisphosphatase (Fbp1) are significantly up-regulated in *C. albicans* biofilm persisters [33]. Glyoxylate cycle is an alternative pathway of TCA cycle, providing precursors for amino acid biosynthesis or carbohydrate biosynthesis [58]. It can be concluded that fungal persisters tend to store energy rather than produce, which help them enter a dormant state (Fig 2). However, current studies on the energy metabolism of fungal persister cells are predominantly conducted in biofilms, related mechanisms may not apply to planktonic cells.

In addition to energy metabolism, changes in protein synthesis also play an important role in the formation of antifungal persistence. In AmB-tolerant *C. albicans* biofilm persisters, conserved protein TMA19 involved in protein synthesis and cell growth, elongation factors EF-1, EF-2, EF-Tu involved in translational elongation, Mcr1, Erg10, Erg13 involved in ergosterol biosynthesis are all down-regulated [33]. Previous studies have shown compromise of cell cycle progression and prolongation of cell life in absence of TMA19 [59,60]. Another study of antibiotic persistence indicated that HipA, a transcription inhibitor, mediates the formation of *Escherichia coli* persisters through phosphorylation of EF-Tu [61], but it has not been reported in fungi. Moreover, in a study of *Saccharomyces cerevisiae*, reducing the induction of the target of rapamycin complex 1(TORC1)-mediated ribosome biosynthesis through Ras pathway can lead to the formation of AmB-tolerant persisters, which is independent of the growth mode and occurs in both biofilm and planktonic. Further, this study extended this mechanism to the clinical pathogenic fungi *C. albicans* and *N. glabratus* [62]. A recent study has found that TORC1 can regulate ribosomal dormancy in an evolutionarily conserved manner by directly targeting a ribosome preservation factor [63]. In general, the biosynthesis of proteins in fungal persisters is downregulated at both the transcriptional and translational levels, which are associated with cellular dormancy (Fig 2).

AmB binds to ergosterol, forming microporous channels on the cell membrane and enabling the drug’s fungicidal activity. Downregulation of ergosterol may therefore reduce the exposure of AmB drug targets [64]. However, an increase in ergosterol production and high expression of associated genes *CYP51A* and *CDR1B* were observed in a study of voriconazole-associated *A. fumigatus* persistence [4]. Considering that azoles work by blocking ergosterol synthesis [65], increased production of ergosterol may help maintain cell membrane integrity, enabling persisters to tolerate voriconazole and survive (Fig 2).

### 2. Stress response pathways protect antifungal persisters to survive

**Fig 3:**
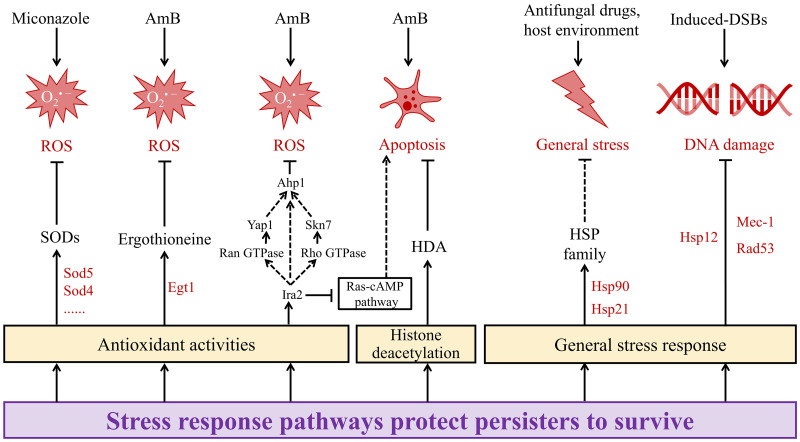
Stress response pathways protect antifungal persisters to survive. When challenged with ROS, persisters up-regulate antioxidant activities, including SODs, ergothioneine, and Ahp1. HDA and Ras-cAMP pathway are involved in the activity against apoptosis, which protect persisters to survive. Moreover, HSP family may participate in some general stress response of persisters, such as Hsp12 in DNA damage. Solid lines: proven in persisters. Dashed lines: likely to occur in persisters. Abbreviations: SODs, superoxide dismutases. Ahp1, alkyl hydroperoxide reductase 1. HDA, histone deacetylase. HSP, heat shock protein. DSBs, double-strand breaks.

Stress response pathways are important for the integrity of fungal cell wall and cell membrane, thus have been widely studied in antifungal tolerance and resistance [66,67]. These pathways are also involved in the formation of antifungal persistence, facilitating the survival of persisters (Fig 3).

Above all, fungal persisters exhibit a powerful antioxidant system to cope with the challenge of ROS produced by drugs or host environment [17]. Bink et al. first found that formation of miconazole-tolerant *C. albicans* biofilm persisters was associated with the ROS detoxification activity of superoxide dismutases (SODs). Except for *SOD2*, all SOD genes were upregulated in miconazole-treated biofilms, with *SOD5* upregulated most. Significantly, the Δ*sod4*/Δ*sod5* double knockout mutant strain has increased ROS level and decreased persistence formation. Moreover, the persister fraction reduced by 18-fold once cells treated with a SOD inhibitor N,N-diethyldithiocarbamate [68]. Furthermore, another anti-oxidative stress enzyme, alkyl hydroperoxide reductase 1 (Ahp1), was found to play a pivotal role in the formation of AmB-tolerant *C. albicans* biofilm persisters. Ahp1 is a member of the peroxiredoxin family, cooperates with other peroxidase such as Trx1 to protect cells from ROS [69]. Studies indicate that Ahp1 can be directly or indirectly regulated by Ira2, an inhibitory regulator of the RAS-cAMP pathway [70]. In *C. albicans*, upregulation of Ras-cAMP-PKA signaling pathway accelerates cell apoptosis [71], but there is no direct evidence that this pathway is involved in the formation of persisters. Moreover, a recent study using a mouse model of pulmonary cryptococcosis found that ergothioneine and its encoding gene, *EGT1*, were significantly overexpressed in AmB persisters. Ergothioneine works synergically with glutathione to enhance tolerance to ROS, thereby supporting persistence in stationary-phase cells [3]. In another study, inhibitors of histone deacetylase (HDA) could enhance the effect of AmB and even eradicate persisters of *C. albicans*. ROS caused by AmB may lead to histone acetylation and activate apoptosis in persisters, highlighting the important role of HDA in protection against apoptosis and persistence formation [32]. In addition to the previously mentioned enzymes and pathways, biofilms formed by persister cells may also serve as a mechanism to combat oxidative stress. It has been found that persister cells’ biofilms had distinct architectural structures and exhibited significantly enhanced antioxidant capacity compared to those formed by normal planktonic cells [72].

Besides ROS, other stress factors are also involved in the formation of antifungal persistence. It has been found that spontaneous DNA damage triggers persistence in *S. cerevisiae* by activating the general stress response, withstanding a series of environmental stress and drug pressure. Moreover, this study also found that Hsp12 marked persisters in budding yeast, because of its high expression in cells that survive under extreme environmental stresses [45]. Hsp12 is part of the environmental stress response and plays a crucial role in protecting cells from severe stress, likely contributing to the formation and maintenance of persistence [73]. In addition to Hsp12, other proteins from the heat shock protein (HSP) family, including Hsp90, Hsp21, Hsp104, HSP Ssc1, and Hsp70 molecular chaperone protein Kar2, are highly expressed in AmB-tolerant persisters [33]. Hsp90 is central to stress response pathways in fungi, mediating antifungal tolerance and resistance through its interactions with key client proteins: protein phosphatase calcineurin and components of the PKC cell wall integrity pathway [66,74]. However, Hsp90 and other heat shock proteins have not yet been directly studied in the context of antifungal persistence.

### 3. Adhesion plays a key role in antifungal persistence formation

Biofilm formation helps fungal cells survive antifungal treatment by secreting extracellular matrix and enhancing drug efflux pump activity, which is closely related to antifungal tolerance and resistance [75–77]. Antifungal persistence is most commonly observed in biofilm rather than in planktonic cells [2,33,34], highlighting the critical role of biofilm formation in antifungal persistence. This is distinctly different from antibiotic persistence, which is mostly detected in planktonic cells [78]. However, LaFleur and colleagues first reported that the biofilm-deficient double mutant strain *efg1*Δ/*cph1*Δ did not result in the reduction of AmB-triggered persisters, indicating that antifungal persistence is not solely dependent on matured biofilm structure formation [2]. A separate investigation demonstrated that not all biofilm-forming *C. albicans* strains necessarily generate persister cells, which supported the aforementioned observation [79]. A following study revealed that persistence formation mainly occurred in the stage of surface adhesion, which is necessary for the emergence and maintenance of AmB-tolerant *C. albicans* biofilm persisters [34]. Moreover, in a study on voriconazole-tolerant *A. fumigatus* persistence, two galactosaminogalactan (GAG) biosynthesis genes, *SPH3* and *UGE3*, were significantly overexpressed in persisters [4]. It has been proven that GAG can directly enhance adhesion of *A. fumigatus* to host cells or material surfaces [80]. Further, *BCR1*, a gene that promotes biofilm formation by regulating adhesion through the major surface adhesins Hwp1 and Als3, exhibits significantly higher expression in persistent *C. albicans* populations compared to non-persistent groups, both with and without AmB induction. Meanwhile, *EFG*1, a gene regulates biofilm development and cell morphology, has no significant difference between the two groups [81]. The above evidence suggests that persister formation is linked to the initial adhesion phase of biofilm formation, rather than later maturation stages. However, current research in this field has been largely limited to investigations of biofilm-associated genes, lacks mechanistic studies elucidating specific pathways, and covers a limited range of fungal species.

## Conclusion and perspectives

This review highlights the clinical significance of antifungal persistence. While current understanding relies heavily on murine models and observational clinical data, conclusive evidence directly tying fungal persistence to clinical outcomes, such as recurrent infections, is still lacking. Addressing this critical knowledge gap represents an urgent priority for future investigations.

The mechanisms underlying antifungal persistence remain poorly defined and appear to be multifactorial. Notably, AmB-tolerant persister cells are absent in biofilms of some *Candida spp.*, suggesting that antifungal persistence is not a common phenomenon [79,82]. Furthermore, experimental induction of dormancy through 5-fluorocytosine-mediated inhibition of nucleic acid and protein synthesis failed to enhance AmB persistence in *C. albicans* biofilm, demonstrating that dormancy alone is insufficient for the persistence development [43]. These findings underscore the need for further investigation into the molecular basis of antifungal persistence.
